# Reproductive coercion and abuse among pregnancy counselling clients in Australia: trends and directions

**DOI:** 10.1186/s12978-022-01479-7

**Published:** 2022-07-30

**Authors:** Nicola Sheeran, Kari Vallury, Leah S. Sharman, Bonney Corbin, Heather Douglas, Brenna Bernardino, Maria Hach, Leanne Coombe, Sophie Keramidopoulos, Regina Torres-Quiazon, Laura Tarzia

**Affiliations:** 1 School of Applied Psychology, Griffith University, Mt Gravatt Campus, 176 Messines ridge road, Mt Gravatt, Brisbane, Australia; 2 Children by Choice, Brisbane, Australia; 3 School of Psychology, University of Queensland, Brisbane, Australia; 4 Marie Stopes Australia, Melbourne, Australia; 5 Melbourne Law School, University of Melbourne, Melbourne, Australia; 6 Multicultural Centre for Women’s Health, Melbourne, Australia; 7 Faculty of Medicine, University of Queensland, Brisbane, Australia; 8 Department of General Practice, University of Melbourne, Melbourne, Australia; 9 Centre for Family Violence Prevention, Royal Women’s Hospital, Melbourne, Australia

**Keywords:** Reproductive coercion, Violence against women, Migrant and refugee women, Aboriginal and/or Torres Strait Islander women, Australia, Sexual and reproductive health

## Abstract

**Background:**

Reproductive coercion and abuse (RCA) interferes with a person’s reproductive autonomy and can be classified into behaviours that are pregnancy promoting or pregnancy preventing (including coerced abortion). However, prevalence data are lacking, and little is known about whether particular forms of RCA are more or less common. The aims of our study were to explore how frequently people seeking pregnancy counselling reported RCA, the proportions reporting the different forms of RCA, and whether there were different trends based on a range of demographic factors.

**Methods:**

Data were collected from 5107 clients seeking counselling support for their pregnancy between January 2018 and December 2020 from two leading providers of pregnancy counselling and sexual and reproductive health services in Australia, Marie Stopes Australia and Children by Choice. Counsellors identified and recorded the presence of RCA and whether the behaviour was pregnancy promoting and/or pregnancy preventing. Demographic factors included age, and whether the person identified as being from a migrant or refugee community or as an Aboriginal and/or Torres Strait Islander person.

**Results:**

RCA was identified in 15.4% of clients, with similar proportions disclosing RCA towards pregnancy (6%) and towards pregnancy prevention or abortion (7.5%), and 1.9% experiencing RCA towards pregnancy and abortion concurrently. There were no differences based on age or whether the person identified as being from a migrant or refugee background, though people who identified as Aboriginal and/or Torres Strait Islander experienced RCA that was significantly more likely to be pregnancy promoting.

**Conclusions:**

RCA is commonly disclosed by people seeking support in a pregnancy counselling context, and coercion and abuse is equally likely to be towards pregnancy promotion or pregnancy prevention/abortion. Given the prevalence and negative impacts of RCA, regardless of age and background, we recommend sensitive and culturally respectful enquiry around experiences of RCA be embedded in healthcare, health education, and health research.

## Plain Language Summary

Reproductive coercion and abuse (RCA) is behaviour that interferes with a person’s decision to become pregnant or to continue a pregnancy. We classified RCA into behaviours that attempt to promote pregnancy or to prevent/end a pregnancy. Drawing on data collected from 5107 people seeking counselling support for their pregnancy from two Australian services, this research explored how common the different types of RCA are. The research also looked at whether a person’s age or whether the person identified as being from a migrant or refugee community or as an Aboriginal and/or Torres Strait Islander person made any difference to the type of RCA they experienced. We found that 15.4% of people reported RCA, with similar proportions reporting behaviours attempting to promote pregnancy and prevent/end pregnancy. Around 2% reported experiencing both forms of RCA. We found that there were no differences in frequency of RCA based on age or whether the person identified as being from a migrant or refugee background, although we found that people who identified as Aboriginal and/or Torres Strait Islander were proportionally more likely to experience RCA that was pregnancy promoting. Given how common RCA is, regardless of age and background, we recommend sensitive and culturally respectful enquiry around experiences of RCA be included in any conversations around sexual and reproductive health care and education.

## Introduction

Reproductive coercion and abuse (RCA) is an often hidden yet increasingly recognised form of interpersonal violence. It involves a range of behaviours intended to interfere with or control an individual’s ability to make autonomous reproductive decisions, most notably to become pregnant or to terminate a pregnancy [1–3]. RCA is often perpetrated by past or current intimate partners, though family members can also be abusers or instigators [2, 4]. RCA is closely associated with other types of coercion and violence in relationships and increases the risk of unintended pregnancies and poor mental health outcomes [5–7]. Despite these negative health outcomes, RCA continues to be a relatively hidden problem in Australia and the limited body of research precludes the development of practice guidelines that might assist health practitioners to respond effectively [1].

**Fig. 1:**
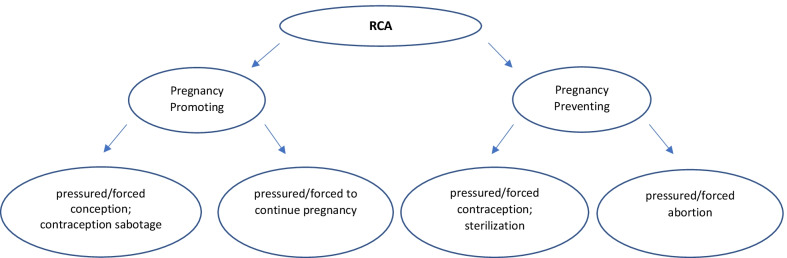
Forms of RCA as a function of whether it is aimed at promoting or preventing pregnancy

RCA, alternatively known as *reproductive coercion* [8] and *reproductive control*, describes a range of interpersonal behaviours that deliberately compromise reproductive autonomy by coercing or forcing a person to become pregnant and/or to continue a pregnancy, to terminate a pregnancy and/or to prevent a pregnancy [3]. While there is ongoing definitional debate around whether structural forms of RCA (i.e., via laws, policies, and social norms) should also be considered [3], in this article, we focus on interpersonal forms of RCA. Similarly, while there is still much debate about what term best captures these behaviours, we use RCA to acknowledge that abusers may use psychological, physical, financial, and sexual violence and not just coercion to influence, control or force compliance. Common behaviours associated with RCA that are pregnancy promoting include contraceptive sabotage, forced sex to cause pregnancy, emotional pressure, threats and/or violence to become pregnant or continue a pregnancy. Common behaviours associated with RCA that are pregnancy preventing include forced contraception use or sterilisation, emotional pressure, threats and/or violence to ensure a pregnancy is terminated, or physical violence to induce a miscarriage [3]. Recent research suggests that although each form of RCA may occur in isolation, victims/survivors can experience multiple forms of RCA within a single pregnancy [9]. Most research to date has considered RCA as a global category, which obscures our understanding of whether there are differences in the prevalence of different forms of RCA and whether the different forms of RCA are associated with unique behaviours (Fig. 1).

International studies indicate that between five and 30% of women may experience RCA in their lifetime [2, 10], although there are problems and inconsistencies in how RCA has been measured within the extant literature [3]. For example, most research relies on self-report data where a limited range of behaviours that constitute RCA are presented, and particular forms of RCA (such as coerced abortion) are often not assessed (i.e., [11]). Further, there is limited published prevalence data to indicate the extent of RCA in Australia. However, Price et al. [7] identified around 6% of clients of a pregnancy counselling service in Queensland reported experiencing RCA while Tarzia et al. found that 9.6% of women recruited in general practice waiting rooms in Victoria reported experiencing contraceptive sabotage and/or coerced pregnancy [12]. To date, no studies have assessed prevalence of RCA in a national Australian sample.

The risk factors for RCA are not entirely clear, though existing research consistently suggests that RCA has strong associations with other forms of domestic violence (DV) [2, 13, 14] and sexual violence (SV) [11, 14–16]. A recent Australian study found that over 20% of women who reported DV while accessing pregnancy counselling, also reported RCA [7]. In terms of demographic risk factors, there are inconsistent and contradictory findings across the literature. Some studies suggest that lower levels of education [8, 17, 18], lower socioeconomic status [19] and being single or in a casual intimate relationship [19] may be significantly associated with RCA. However, this evidence is limited, and may depend on how RCA is measured in survey instruments [3]. Age also seems to be a factor, with some studies suggesting that younger age is positively associated with RCA [16, 17] and others finding the opposite [18].

Additionally, there is a dearth of research that explores the experiences RCA among people from racialized and/or marginalised communities, including migrant and refugee communities. International research on the relationship between RCA and race and/or cultural identity has reported mixed findings, with some studies suggesting an increased risk of RCA among self-identified Black, Hispanic, and mixed-race women, and others not [17, 19–21]. Again, however, research on this relationship is plagued with measurement issues, and has generally lacked a nuanced consideration of the intersections between RCA, race and/or cultural identity, marginalisation, and structural inequality. In the Australian context, little quantitative research has been done to explore the link between race and/or cultural identity and RCA, although Price et al. [7] found that the prevalence of RCA and co-occurring DV was higher for people who identified as Aboriginal and/or Torres Strait Islander. Qualitative work by Griffiths et al. [22] suggests that women in some Aboriginal communities may experience pressure to become pregnant, highlighting the need to better understand the prevalence and implications of RCA in Aboriginal and Torres Strait Islander communities. Tarzia et al. [23] reported qualitative data from specialist and legal practitioners in Australia that suggested the intersection between structural vulnerabilities and men’s violence may place migrant and refugee women at increased risk of RCA, but again, no quantitative data currently supports this.

In light of the research gaps described above, this study aimed to elucidate patterns of RCA, including the proportions of different forms of RCA among people who were accessing counselling regarding their pregnancy across Australia. Specifically, the study sought to address whether (a) people seeking counselling for their pregnancy who report experiencing RCA more commonly experience coercion that is pregnancy promoting or pregnancy preventing (i.e., coercion towards abortion); and (b) whether these trends differ by demographic factors such as age and whether the person identified as being from a migrant or refugee community or as an Aboriginal and/or Torres Strait Islander person.

## Methods

### Study context

Marie Stopes Australia (MSA) is a national not-for-profit provider of sexual and reproductive health services across 17 locations, including 14 clinics and a national telehealth service. Each year the organization offers over 50,000 clinical services including contraception care, abortion care, vasectomy, tubal ligation, and sexually transmitted infection testing. Some clinical services are entirely available via telehealth whilst others may have pre-care or after-care offered via phone or video and require in-person clinic examinations, screens, or procedures. Counselling services are a combination of in-clinic care and telehealth, depending on the location and complexity of care.

Children by Choice (C by C) is an independent, Brisbane-based not-for-profit organisation providing counselling and decision-making support, information and referrals for women and pregnant people in Queensland, along with post-abortion counselling. These state-wide services are provided online via their website and email, by phone, and in-person in their Brisbane office.

Counsellors at both services had undertaken, designed and/or delivered training in RCA screening, and worked for two of the leading voices in RCA in Australia (see [1, 24]). For example, C by C provide training on RCA nationally and MSA produced the seminal report on RCA in Australia, which meant that staff working in the services had a thorough understanding of behaviours that constitute RCA. This likely meant that a broader range of behaviours were captured than in previous research.

### Participants and procedure

The study included 5107 people who had contacted the counselling services at Marie Stopes Australia (n = 3109) or C by C (n = 1998) for support pertaining to their current, and in some instances a past, pregnancy. Support could include pregnancy decision making counselling, post-abortion counselling or information about pregnancy options. We employed a total sampling strategy whereby all clients who were currently living in Australia and contacted MSA for pregnancy options counselling pertaining to a current unplanned pregnancy between January 2018 and June 2020 and all clients who contacted C by C for pregnancy decision-making or post-abortion counselling, or information and referral pertaining to a pregnancy between October 2018 and July 2020, were included in the study. Thus, those accessing MSA were currently pregnant but those accessing C by C were either currently or had recently been pregnant.

The average age of participants was 29.19 years (*SD* = 7.08, *r* = 13–50 years) and most participants contacted the service once (59.5%) or twice (21%), though the range was large (1–65 contacts). The sample consisted of participants who identified as being from a migrant or refugee (n = 1162) community and people who identified as Aboriginal and/or Torres Strait Islander (n = 283). Table 1 provides demographic data of the sample for those who were had experienced reproductive coercion and those who had not.

**Table 1:** Demographic details of the sample for those experiencing and not experiencing reproductive coercion

	Reproductive coercion present N = 782	No reproductive coercion present/not asked N = 4325
Mean age (SD) years	28.56 (6.80)	29.32 (7.12)
Identified as aboriginal and/or Torres Strait Islander	N = 52	N = 226
Identified as being from a migrant or refugee community	N = 189	N = 973
Mean number of times contacting the service (SD)	2.78 (4.13)	1.86 (1.82)

Ninety-eight percent of participants from Children by Choice were from Queensland, while participants from Marie Stopes Australia were most commonly from Victoria and New South Wales (see Table 2). Notably, small numbers of participants were from South Australia, which is likely due to the service delivery model in that state.

**Table 2:** Percentage of participants accessing C by C and MSA counselling services during 2018–2020 by Australian state

	ACT N (%)	NSW N (%)	NT N (%)	QLD N (%)	SA N (%)	TAS N (%)	VIC N (%)	WA N (%)	TOTAL N
MSA	152 (4.9)	1032 (33.2)	16 (0.5)	440 (14.2)	5 (0.2)	21 (0.7)	1010 (32.5)	426 (13.7)	3102
C by C	0	23 (1.2)	2 (0.1)	1962 (98)	2 (0.1)	3 (0.2)	4 (0.2)	2 (0.1)	1998
Total	152 (3)	1055 (20.6)	18 (0.4)	2402 (47)	7 (0.1)	24 (0.5)	1014 (20)	428 (8.4)	5100

*ACT* Australian Capital Territory; *NSW* New South Wales; *NT* Northern Territory; *QLD* Queensland; *SA* South Australia; *TAS* Tasmania; *VIC* Victoria; *WA* Western Australia; *MSA* Marie Stopes Australia; *C by C* Children by Choice

### Data collection

Data included in the current study was collected as part of routine data collection by the services. Both services routinely record information about RCA from clients during their contact with the services. While both services collect a range of information from clients, there were only a small number of fields that were consistently collected by both services and that were able to be input into this combined analysis. These fields included age, location, whether the person was from a migrant and refugee background (recorded by C by C as culturally and linguistically diverse and/or refugee) or identified as being an Aboriginal and/or Torres Strait Islander person, whether the person has experienced RCA, and if so, whether the coercion was towards continuing with a pregnancy, towards abortion, or both.

Both services employ a sensitive inquiry approach to broaching RCA whereby questions about abuse are asked sensitively and as appropriate during the consultation. If, during the contact with the service, the client reported behaviours that were consistent with RCA, the counsellor would select checkboxes to indicate that RCA in various forms was present. Multiple boxes could be selected if various forms of RCA were present. RCA that promoted pregnancy included behaviours that had resulted in the person becoming pregnant (i.e., incessant pressure to be pregnant, psychological, or physical harm or threats if does not get pregnant or refuses sex, forced sex causing pregnancy, or contraception sabotage), as well as pressure or coercion to continue with the pregnancy. RCA that prevented pregnancy/promoted abortion included emotional blackmail, threats, pressure, or coercion to terminate the pregnancy, or physical violence to induce miscarriage. Binary variables were created from the various checkboxes collected by each organisation as the presence of RCA (yes or no/not asked), coercion and abuse that was pregnancy promoting (yes or no/not asked) and coercion and abuse that was pregnancy preventing/promoted abortion (yes or no/not asked).

Much of the research on RCA has relied on self-report data where a range of behaviours are presented, and participants indicate whether they have experienced the behaviours [17, 25]. One limitation has been the often-narrow range of behaviours that have been included, including a lack of questions that measured coercion toward abortion [6, 11]. However, our understanding of RCA is continuously growing along with our knowledge of the tactics or behaviours perpetrators may employ [3]. We sought to overcome this in the current study by using counsellor identified behaviours that were consistent with RCA. This likely meant that a broader and more comprehensive range of behaviours were captured than in previous research.

### Analyses

We undertook secondary analysis of the data routinely collected by C by C and MSA. Given our data set consisted of only categorial data, Chi Square analyses were used to examine whether the observed frequencies of RCA found in the data were statistically significantly different (p < 0.05) to what would be expected for each group of participants. To explore whether the pattern of RCA differed by age, we calculated the frequency of RCA across common age brackets.

## Results

As shown in Table 3, 15.4% of participants (n = 783) reported experiencing some form of RCA and the proportion of participants reporting coercion that was pregnancy promoting and preventing was similar. Of the total sample, 1.9% (n = 97) reported that they had experienced RCA that was both pregnancy promoting and preventing concurrently. However, considering only those who were experiencing some form of RCA, 20.3% (n = 97/N = 382) of those who experienced coercion that was pregnancy preventing/toward abortion and 24.2% (n = 97/N = 304) of those who experienced coercion that was pregnancy promoting reported experiencing both forms of coercion.

**Table 3:** Proportion of participants of participants accessing C by C and MSA counselling services during 2018–2020 identified as experiencing RCA by cultural background

	Reproductive coercion present			No/not asked	Total
	RCA that promoted pregnancy only N (%)	RCA that was pregnancy preventing/abortion only N (%)	Experiencing both forms of RCA N (%)	N (%)	N (% of total)
Total sample	304 (6.0)	382 (7.5)	97 (1.9)	4324 (84.7)	5107
Migrant/Refugee	83^a^ (7.1)	87^a^ (7.5)	19 (1.6)	973 (83.7)	1162 (22.8)
Aboriginal and/or Torres Strait Islander	26^a^ (9.1)	18^b^ (6.3)	8 (2.8)	231 (81.6)	283 (5.5)
MSA	157^b^ (4.9)	276^a^ (8.5)	53 (1.6)	2780 (85.1)	3109 (60.9)
C by C	147^a^ (7.4)	106^b^ (5.3)	44 (2.2)	1701 (85.1)	1998 (39.1)

Percentages represent percentages of participants from that organisation or identifying as a migrant or refugee/Aboriginal and/or Torres Strait Islander. Columns with the same letter are not significantly different from each other at p < .05. Columns with different letters are significantly different from each other at the P < .05

*MSA* Marie Stopes Australia; *C by C* Children by Choice

To check the equivalency of our samples from each of the services, we compared the frequency of RCA for MSA and C by C finding no differences between the proportion of participants reporting coercion from each of the services, χ^2^(1, N = 5107) = 0.458, p = 0.458, with overall rates almost identical (C by C = 14.9%; MSA = 15%). However, there were significant differences between the proportion of participants reporting coercion that was pregnancy promoting and coercion towards abortion/pregnancy prevention from each of the services, χ^2^(1, N = 5107) = 13.226, p < 0.001 and χ^2^(1, N = 5107) = 13.529, p < 0.001, respectively. Specifically, more participants accessing MSA reported coercion towards abortion/pregnancy prevention than coercion that promoted pregnancy. Conversely, more participants accessing C by C reported coercion that promoted pregnancy than towards abortion/pregnancy prevention (see Table 3).

No significant differences in the presence of RCA were found between the proportion of participants who identified as being a migrant or refugee compared to those who did not identify as a migrant or refugee, χ^2^(1, N = 5107) = 1.009, p = 0.315. Similarly, there were no differences between the proportion of participants reporting coercion that promoted pregnancy who identified as a migrant or refugee and those who did not, χ^2^(1, N = 5107) = 1.783, p = 0.182 and no differences between the proportion of participants reporting coercion towards abortion/pregnancy prevention who identified as a migrant or refugee and those who did not, χ^2^(1, N = 5107) = 0.117, p = 0.732. Together, these findings suggest that people who identify as migrant and/or refugees are no more likely to experience coercion that promoted or prevented pregnancy.

No significant differences in the presence of RCA were found between the proportion of participants reporting reproductive coercion who identified as Aboriginal and/or Torres Strait Islander and those who did not, χ^2^(1, N = 4950) = 3.487, p = 0.062. Similarly, there were no differences between the proportion of participants reporting coercion towards abortion/pregnancy prevention who identified as Aboriginal and Torres Strait Islander and those who did not, χ^2^(1, N = 5107) = 0.000, p = 0.987. However, there were significant differences between the proportion of participants reporting coercion that promoted pregnancy who identified as Aboriginal and/or Torres Strait Islander and those who did not, χ^2^(1, N = 5107) = 7.789, p = 0.005, suggesting that those who identified as Aboriginal and Torres Strait Islander more frequently experienced coercion that promoted pregnancy.

No significant differences in the presence of reproductive coercion were found between participants across different ages, χ^2^(7, N = 4544) = 12.105, p = 0.097. Similarly, there were no significant differences in the proportion of participants reporting coercion that promoted pregnancy at across different ages, χ^2^(7, N = 4544) = 13.193, p = 0.068, nor were there differences in the proportion of participants experiencing coercion towards abortion/pregnancy prevention across different ages, χ^2^(7, N = 4544) = 8.314, p = 0.306. A logistic regression analysis also indicated a non-significant association between age and RCA (χ^2^(7, N = 4544) = 12.772, p = 0.078) with age accounting for less than 1 percent of the variation. Together these suggest that no particular age group (within the subset of women of reproductive age) is more likely to experience coercion that promoted or prevented pregnancy (see Table 4).

**Table 4:** Proportion of participants accessing C by C and MSA counselling services during 2018–2020 identified as experiencing RCA by age

Age (years)	Reproductive coercion present (n = 783)				No/not asked N (%)
	N (%)	RCA that promoted pregnancy N (%)	RCA that prevented pregnancy/abortion N (%)	Both N (%)	
≤ 13	4 (0.1)	1 (0.3)	0 (0)	0 (0)	3 (0.07)
14–18	240 (4.6)	9 (2.9)	20 (5.2)	6 (6.1)	205 (4.92)
19–24	1081 (21.2)	69 (22.7)	97 (25.4)	28 (28.9)	887 (21.3)
25–29	1047 (20.5)	79 (25.9)	76 (19.9)	27 (27.8)	865 (20.7)
30–34	980 (19.2)	57 (18.8)	81 (21.2)	17 (17.5)	825 (19.8)
35–39	839 (16.4)	50 (16.4)	66 (17.2)	11 (11.3)	712 (17.1)
40–44	318 (6.2)	14 (4.6)	18 (4.7)	4 (4.1)	282 (6.7)
≥ 45	35 (0.6)	1 (0.3)	1 (0.2)	1 (1)	32 (0.7)
Missing age	563 (11.2)	24 (7.8)	23 (6)	3 (3)	513 (12.3)
Total	5107	304	382	97	4167

Percentage in brackets is a percentage of column total

## Discussion

The aim of this study was to elucidate the patterns of RCA and whether RCA was more frequently aimed at pregnancy promotion or pregnancy prevention/abortion. We found no difference in overall rates of RCA across the services with RCA identified as being experienced by around 15% of clients at both MSA and C by C. Overall, the rates of coercion that promoted and prevented pregnancy were also similar. Much of the previous research focuses on forced pregnancy and condom sabotage, which effectively excludes half of those experiencing RCA and makes prevalence appear lower. In particular, a large body of research draws on the National Intimate Partner and Sexual Violence survey conducted in the United States of America [26], which only includes pregnancy coercion and condom refusal when measuring RCA. Conclusions drawn from this data pertaining to racial differences (i.e., [11]) may be misrepresenting the scope and complexity of the issue experienced by different cultural and racial groups.

Interestingly, we found that 2% of the sample reported experiencing both coercion towards abortion and coercion that was pregnancy promoting. Scant attention has been paid to what forms RCA takes within and across different pregnancies and our findings suggest that people may be contending with coercive and abusive behaviours that are contradictory. More research is needed to understand whether the different types of RCA were perpetrated by the same person or different people and also the temporal pattern of the different forms of RCA. If it is perpetrated by the same person, it raises interesting questions about the role of intent and what this looks like. For example, the pattern of RCA may mirror patterns of coercive control, where the tactics used to assert control may change over time (i.e., sexual assault to promote pregnancy and then coercion or abuse to promote or induce abortion), based on the abuser’s knowledge of the victim/survivor, and where the victim/survivor feels they are walking on eggshells as the rules change [27]. It could also be that, as with sexual violence [28], those who have previously experienced particular forms of RCA may be at risk of experiencing them again. Alternatively, there may be different abusers where a person/people are using coercion or abuse to force one decision while another person is forcing a different one (i.e., a young person whose parents are coercing one decision or extended family in some communities may be more likely to coerce pregnancy and the man involved in the pregnancy using coercion to force a different decision).

Another key finding was that people who identified as Aboriginal and/or Torres Strait Islander were more likely to experience coercion that promoted pregnancy than coercion towards abortion/pregnancy prevention. Rather than speculate on why this might be, we instead argue that further research is warranted to understand reproductive autonomy and what that means to Aboriginal and/or Torres Strait Islander peoples, including facilitators and barriers to reproductive autonomy. For example, Griffiths et al. [22] highlighted a complex interplay between reproductive autonomy, modern contraceptive use, and traditional cultural practices for Aboriginal women in Western Australia and emphasized the need for respectful and inclusive reproductive health services. Exploring the root causes of coercion that promotes pregnancy may lead to greater cultural safety and improved health outcomes for Aboriginal and/or Torres Strait Islander peoples.

We also found that there were no significant differences in the experience of the different forms of RCA for people from migrant and refugee backgrounds nor across different age groups. This is an important finding that highlights the need to recognise that clients of any age and background could be experiencing RCA and to remember to sensitively enquire, as well as to provide culturally appropriate education materials, health system support, and interventions across the reproductive lifespan.

### Practical implications

In Australia, family, domestic and sexual violence is under-reported, and barriers to accessing a full suite of sexual and reproductive health services (including abortion care) and help-seeking for victims/survivors of violence persist ([29] [AIHW], [30]). These are particularly salient for people experiencing intersecting disadvantages, including those who live in under-resourced areas outside of urban centres [31, 32]. RCA inherently further exacerbates challenges victim/survivors face in accessing the services they need to facilitate autonomous reproductive decision making, compounding accessibility issues for those who experience other structural, geographic or financial barriers. The identification of RCA may be useful as a ‘soft entry’ to identifying further family, domestic or sexual violence, given their co-occurrence, and crucial to ensuring victim-survivors accessing sexual and reproductive health services are able to make autonomous reproductive health decisions. Therefore, understanding, enquiry for, and workforce training around RCA is ever more crucial to ensure the realisation of reproductive health, rights, and justice for all. Recently, interviews with primary care clinicians around Australia identified a lack of awareness of, structural and practical support for [33], and confidence in identifying or responding to RCA in primary care settings. Similarly, “reproductive coercion” was a relatively new term for workers in domestic violence services, who tended to define it very broadly [34]. There are indications that coercive control and violence, lack of culturally responsive service delivery, and structural barriers to essential health care and support, compound to make some women and pregnant people particularly vulnerable, and these sorts of intersections warrant further research attention [23, 35].

### Limitations and future directions

The current study diverged from most of the extant literature by measuring prevalence based on counsellor-identified RCA rather than relying on self-report data. This was in part an opportunistic decision based on the availability of routine practice data from our project partners; yet, it should also be noted that this method may have some advantages. These include allowing a broader range of behaviours to be identified compared to a measure with limited items that may not capture the complexity or scope of RCA. The counsellors at both MSA and C by C had received training around RCA and were likely to have a good understanding of what behaviours constitute it. On the other hand, it is also possible that counsellors were too broad or too inclusive. There is some controversy around when and at what point ‘pressure’ is considered coercion. While papers such as Tarzia and Hegarty [3] for example, have centred intent, fear, and control as fundamental components of RCA that differentiate it from other behaviours, we have no knowledge of whether these were assessed by counsellors in all instances. Conversely, RCA may not have been disclosed during the counselling session/s, which would mean that our findings are conservative. Future research is needed to improve measurement of RCA including the development of standardized, evidence-based screening procedures and training for providers. This will improve both data collection and identification of women and pregnant people in need of support.

Further, depending on the length of contact with the client, demographic factors may or may not have been collected, meaning that our study may be under representative of those from migrant and refugee and Aboriginal and/or Torres Strait Islander communities. We also utilised a crude measure of a person’s racial and/or ethnic identity and did not collect more accurate data (i.e., whether they were particular visa holders, how long they have been in Australia, place of birth, etc.) or broader intersecting categories of identity (e.g., disability, gender or gender diversity, sexuality or intersex status). Future research is warranted to explore the intersectional experiences of RCA across Australia. While the sample included participants from all Australian states, it was not nationally representative, with Queensland overrepresented and South Australia underrepresented, most likely due to the legal and policy contexts and service agreements for pregnancy options counselling and abortion provision in those states. Thus, our findings may only be representative of clients who choose to access pregnancy decision making counselling or post-abortion support from these particular services. Finally, our study was descriptive in nature and the field would benefit from more rigorous assessment of RCA and its antecedents and consequences.

## Conclusion

Despite the limitations of our data, our study is the first to investigate prevalence of RCA nationally in Australia and suggests that around 15% of those seeking counselling support following an unplanned pregnancy experience RCA. Further, we found that coercion toward abortion and continuing with a pregnancy are equally prevalent, though the proportion of people experiencing both types of RCA warrants further exploration. As age and whether a person is from a migrant or refugee community or Aboriginal and/or Torres Strait Islander do not meaningfully predict RCA, we recommend sensitive enquiry and culturally safe approaches to experiences of RCA be embedded in all health care, health education, and health research.

## Data Availability

The data that support the findings of this study are available from Marie Stopes Australia and Children by Choice, but restrictions apply to the availability of these data, which were used under license for the current study, and so are not publicly available. Data are however available from the authors upon reasonable request and with permission of Marie Stopes Australia and Children by Choice.

## Abbreviations

RCA: Reproductive coercion and abuse
MSA: Marie Stopes Australia
C byC: Children by Choice
DV: Domestic violence
SV: Sexual violence
